# Comparing Flaxseed and Perindopril in the Prevention of Doxorubicin and Trastuzumab-Induced Cardiotoxicity in C57Bl/6 Mice

**DOI:** 10.3390/curroncol29050241

**Published:** 2022-04-21

**Authors:** Cameron R. Eekhoudt, Tessa Bortoluzzi, Sonu S. Varghese, David Y. C. Cheung, Simon Christie, Skyler Eastman, Ishika Mittal, J. Alejandro Austria, Harold M. Aukema, Amir Ravandi, James Thliveris, Pawan K. Singal, Davinder S. Jassal

**Affiliations:** 1 Institute of Cardiovascular Sciences, Department of Physiology and Pathophysiology, Max Rady College of Medicine, Rady Faculty of Health Sciences, University of Manitoba, 432 Basic Medical Sciences Building, 745 Bannatyne Avenue, Winnipeg, MB R3E 0J9, Canada; ceekhoud@student.ubc.ca (C.R.E.); bortolut@myumanitoba.ca (T.B.); varghes1@myumanitoba.ca (S.S.V.); dcheung@sbrc.ca (D.Y.C.C.); eastman4@myumanitoba.ca (S.E.); ishika_mittal@hotmail.com (I.M.); aaustria@sbrc.ca (J.A.A.); aravandi@sbgh.mb.ca (A.R.); psingal@sbrc.ca (P.K.S.); 2 Section of Cardiology, Department of Internal Medicine, Max Rady College of Medicine, Rady Faculty of Health Sciences, University of Manitoba, Room GC430, Health Sciences Centre 820 Sherbrook Street, Winnipeg, MB R3A 1R9, Canada; umchris8@myumanitoba.ca; 3 Canadian Centre for Agri-Food Research in Health and Medicine, Department of Food and Human Nutritional Sciences, University of Manitoba, Room W573 Duff Roblin Building, Winnipeg, MB R3T 2N2, Canada; harold.aukema@umanitoba.ca; 4 Department of Human Anatomy and Cell Sciences, Max Rady College of Medicine, Rady Faculty of Health Sciences, University of Manitoba, 130 Basic Medical Science Building, 745 Bannatyne Avenue, Winnipeg, MB R3E 0J9, Canada; james.thliveris@gmail.com; 5 Department of Radiology, Max Rady College of Medicine, Rady Faculty of Health Sciences, University of Manitoba, Room GA216, 820 Sherbrook Street, Winnipeg, MB R3T 2N2, Canada

**Keywords:** breast cancer, cardiotoxicity, flaxseed, perindopril, prevention, cardiovascular remodeling

## Abstract

Background: Two anti-cancer agents, doxorubicin (DOX) and trastuzumab (TRZ), are commonly used in the management of breast cancer in women. Despite their efficacy in reducing the morbidity and mortality of individuals with breast cancer, the use of these agents is limited by adverse cardiotoxic side effects. Both the nutraceutical agent flaxseed (FLX) and the pharmaceutical drug perindopril (PER) have been studied individually in the prevention of chemotherapy-mediated cardiac dysfunction. The objective of this study was to determine whether the prophylactic administration of FLX is comparable and/or synergistic with PER in preventing DOX + TRZ-induced cardiotoxicity. Methods: Over a six-week period, 81 wild-type C57Bl/6 female mice (8–12 weeks old) were randomized to receive regular chow (RC) or 10% FLX-supplemented diets with or without PER (3 mg/kg/week; oral gavage). Starting at week 4, mice were randomized to receive a weekly injection of saline or DOX (8 mg/kg) + TRZ (3 mg/kg). Serial echocardiography was conducted weekly and histological and biochemical analyses were performed at the end of the study. Results: In mice treated with RC + DOX + TRZ, left ventricular ejection (LVEF) decreased from 75 ± 2% at baseline to 37 ± 3% at week 6. However, prophylactic treatment with either FLX, PER, or FLX + PER partially preserved left ventricular systolic function with LVEF values of 61 ± 2%, 62 ± 2%, and 64 ± 2%, respectively. The administration of FLX, PER, or FLX + PER was also partially cardioprotective in preserving cardiomyocyte integrity and attenuating the expression of the inflammatory biomarker NF-κB due to DOX + TRZ administration. Conclusion: FLX was equivalent to PER at preventing DOX + TRZ-induced cardiotoxicity in a chronic in vivo murine model.

## 1. Introduction

The leading cause of cancer-related morbidity and mortality in North America is breast cancer [1]. Although anthracyclines, including doxorubicin (DOX), have been an effective mainstay therapy for the treatment of breast cancer, the clinical use of DOX is limited by its cumulative, dose-dependent cardiotoxicity [2,3,4,5,6,7,8,9,10]. Trastuzumab (TRZ), a monoclonal antibody targeted to the extracellular domain of the human epidermal growth factor receptor 2 (HER2) protein, is used in the treatment of HER2-positive breast cancer [3,10,11]. Despite the improvement in overall survival, the use of this monoclonal antibody potentiates the cardiotoxicity associated with DOX [4,10,12]. These significant clinical side effects necessitate the urgency to investigate novel preventative strategies in the setting of DOX + TRZ-mediated cardiotoxicity for maintaining the long-term health of women with breast cancer.

The intricate overlap between oncologic management and cardiovascular side effects has effectively led to the evolving field of cardio-oncology. Both pharmaceutical and nutraceutical products have been efficacious in preventing cardiotoxicity associated with chemotherapy. Numerous studies have demonstrated the prophylactic role for renin-angiotensin system (RAS) antagonists in the prevention of chemotherapy-induced cardiotoxicity [9,13,14,15,16,17]. In the PRADA, MANTICORE 101, and CECCY trials [18,19,20], prophylactic treatment with RAS antagonists provided partial cardioprotection in women with breast cancer treated with anthracycline-based chemotherapy. When comparing the various cardiovascular drugs used in heart failure management, a recent meta-analysis by Ghasemi et al. [21] concluded that ACE inhibitors are superior to other conventional cardiovascular drugs at preserving left ventricular systolic function during anthracycline therapy.

There has been an increasing focus on the use of nutraceuticals as well in the setting of chemotherapy-mediated cardiotoxicity. The current literature suggests that women with breast cancer are using supplements including flaxseed (FLX) as a part of their clinical management [22]. Rich in alpha-linolenic acid and lignans, FLX possesses various anti-inflammatory and anti-oxidative properties [23]. Recently, Asselin et al. [8] demonstrated the cardioprotective role of dietary FLX in preventing adverse cardiovascular remodeling in a chronic in vivo female murine model of DOX + TRZ-mediated cardiotoxicity by reducing inflammation and apoptosis. Although the literature demonstrates the cardioprotective efficacy of PER and FLX individually, little is known regarding the potential prophylactic role of the combination of FLX and PER in the prevention of DOX + TRZ-mediated cardiotoxicity.

The objective of the current study was to determine whether the prophylactic administration of FLX is comparable and/or synergistic with PER in the prevention of DOX + TRZ-mediated cardiotoxicity in a chronic in vivo female murine model.

## 2. Methods

### 2.1. Experimental Animal Model

**Figure 1:**
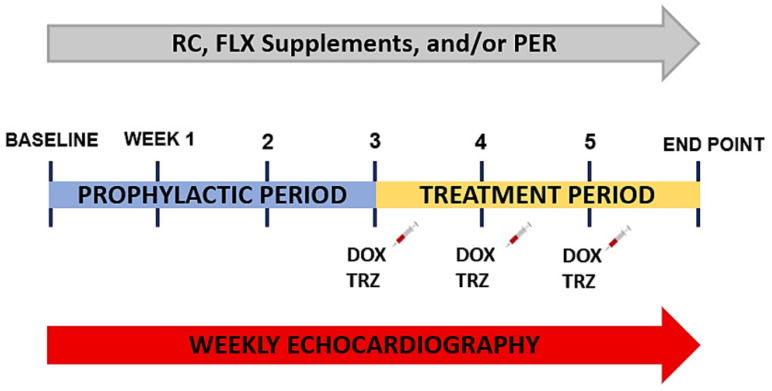
Experimental scheme. Mice received *ad libitum* access to their RC- or FLX-supplemented diets daily starting at baseline for the entirety of the 6-week study. At the start of week 4, mice were further randomized to receive weekly intraperitoneal injections of 0.9% saline or DOX (8 mg/kg) + TRZ (3 mg/kg) to induce a chronic state of chemotherapy-induced cardiotoxicity. Cardiac function was assessed weekly using non-invasive echocardiography. At study endpoint, cardiac tissues were harvested for both histological and biochemical analysis. DOX, doxorubicin; FLX, flaxseed; PER, perindopril; RC, regular chow; TRZ, trastuzumab.

Animal procedures were executed in agreement with the guidelines of the Canadian Council on Animal Care. All procedures were approved by the Animal Protocol Review Committee at the University of Manitoba (REB: 17-022/3 (AC11285)). The study design consisted of a six-week study, composed of a three-week prophylactic period and a three-week treatment period. A total of 81 wild-type C57Bl/6 female mice (8–12 weeks old; Jackson Laboratories, Bar Harbor, ME, USA) were randomly assigned to receive either regular chow (RC) (no. 9GR2, Prolab RMH 3000; TestDiet, St. Louis, MO, USA), 10% FLX-supplemented diets (no. 9GR0, modified Prolab RMH 3000 grain-based semipurified, TestDiet), and/or PER (3 mg/kg) daily for a total of 6 weeks (Figure 1). FLX-supplemented study diets were prepared by TestDiet manufacturing, as previously described [8]. Briefly, 10% FLX was added to modified rodent LabDiet Rat/Mouse/Hamster 3000 with fat sources removed. Diets were assessed and changed for each animal (5 g pellet diet/day) once per week.

After the 3-week prophylactic period, mice were randomized to receive 3 weekly intraperitoneal injections of: (i) 0.9% saline or (ii) DOX (8 mg/kg/week) + TRZ (3 mg/kg/week) to produce a murine model of chronic cardiac dysfunction caused by chemotherapy (Figure 1) [5,6,8,9,24]. The 5 main study groups included RC + saline (*n* = 16), RC + DOX + TRZ (*n* = 20), FLX + DOX + TRZ (*n* = 18), PER + DOX + TRZ (*n* = 10), and FLX + PER + DOX + TRZ (*n* = 17). The administration schedule of DOX (24 mg/kg; Doxorubicin, Pfizer, Kirkland, QC, Canada), TRZ (9 mg/kg; Herceptin, Hoffman-La Roche, Mississauga, ON, Canada), and PER (9 mg/kg; Coversyl, Servier, Laval, QC, Canada) in our chronic in vivo murine model was to recapitulate the dosage used in the clinical setting that has been validated by our group and others [8,24].

Throughout the 6-week study, mice had *ad libitum* access to water and RC and 10% FLX-supplemented diets. All animals were maintained on a 12 h day/night cycle throughout the 6-week period. At the end of the six weeks, mice were euthanized by an intraperitoneal injection of 110 mg/kg of pentobarbital buffered with 2% lidocaine. At study endpoint, cardiac tissue was used for histological and biochemical analyses, including for Western blot analysis. In addition, blood samples were obtained for plasma oxylipin analysis. Plasma was prepared by adding 300 μL of cardiac blood into a Microvette CB 300 300 tube coated with K2EDTA anticoagulant (Cat. # NC9141704: SARSTEDT Inc., Waltham, MA, USA) and centrifuged at 2000× *g* relative centrifugal force for 5 min. Plasma was then collected and stored at −80 degrees Celsius until analyses.

### 2.2. Murine Echocardiography

Murine cardiac function was assessed at baseline and weekly until the end of the 6-week study using transthoracic echocardiography. As previously described [5,6,8,9], a 13 MHz linear array ultrasound probe (Vivid 7, version 11.2, GE Medical Systems, Milwaukee, WI, USA) was used to obtain all images. The EchoPAC PC software (Vivid 7, version 11.2, GE Medical Systems, Milwaukee, WI, USA) was used for offline post-processing of all images. Two blinded observers (CRE and DSJ) completed echocardiographic evaluation.

Evaluation of intra- and inter-observer variability of LV cavity dimensions and function was performed for 30 randomly selected images. To assess intra-observer variability, measurements for random images were performed by a single trained observer (DSJ) on two separate days, two weeks apart. Evaluation of inter-observer variability was assessed by two independent trained observers (CRE and DSJ). Variability was defined as the difference between the two independent observations divided by the mean of the observations and expressed as absolute values.

### 2.3. Histological Analysis

For the ultrastructural analysis, hearts from each study group were processed for electron microscopy as previously described [5,6,8]. For 3 h, cardiac tissue samples harvested from the left ventricle were fixed in 3% glutaraldehyde mixed with 0.1 M phosphate buffer (pH 7.3). Overnight, samples were rinsed in 0.1 M phosphate buffer containing 5% sucrose at 4 °C. For 2 h at room temperature, post-fixation was performed with 1% osmium tetroxide in 0.1 M phosphate buffer and embedded in epon. Cardiac tissue sections were stained with uranyl acetate and lead citrate and subsequently imaged using an electron microscope (CM10 Transmission Electron Microscope, Philips). All images were coded without prior knowledge of the sample study group to prevent observer bias.

### 2.4. Oxylipin Analysis

Plasma oxylipins analysis was done in a subset of mice within each treatment group, as previously described [25,26,27]. A mixture of 100 μL of plasma was added to 1 mL of water at pH 3.0, and 100 μL of internal standard was centrifuged at 14,000× *g* for 10 min at 4 °C to remove debris. Strata-X SPE (33 μL, 60 mg/3 mL, Phenomenex, Torrance, CA, USA) columns were used to extract total plasma oxylipins. Individual oxylipin metabolites were analyzed using HPLC electrospray ionization mass spectroscopy, as previously described [25,26,27].

### 2.5. Western Blotting

Total protein extraction from heart tissue was performed on a subset of mice from each group and subsequently quantified by use of the Bradford assay, as previously described [8]. Sodium dodecyl sulfate polyacrylamide gel electrophoresis was employed at 55 mA for 120 min to separate 30 μg of protein and subsequently transferred to a polyvinylidene fluoride membrane (Product #: 288520, Thermo Scientific, Waltham, MA, USA) for 1 h at 100 volts. The membranes were blocked for 60 min and probed overnight at 4 °C with a 1:1000 dilution of primary antibody specific to a key inflammatory marker NF-κB (Product #: 8242S, Cell Signaling Technology, Danvers, MA, USA). Finally, membranes were probed for 60 min with a 1:5000 dilution of horseradish peroxidase-conjugated goat anti-rabbit secondary antibody (Product #:170-6515, BioRad, Heracles, CA, USA) and detected on CL-Xposure blue X-ray film (Product #: XC6A2, Mandel Scientific Company Inc., Guelph, ON, Canada). GAPDH (protein loading control) was detected using a 1:10,000 dilution of anti-GAPDH antibody (Product #:2118 L, Cell Signaling Technology). Densitometric analysis was performed to assess protein expression normalized to a GAPDH using QuantityOne software (BioRad).

### 2.6. Statistical Analysis

All data are expressed as mean ± standard deviation. The data for Western analysis are expressed as mean ± standard error mean. Repeated measures of one-way ANOVA were used to determine significance between independent factors for post hoc analysis. The *p* values for main effects and interactions were noted when appropriate. Mann–Whitney and Kruskal–Wallis tests were applied in histological analysis for non-parametric comparison of scores, as previously described [8,9]. ANOVA with Dunnett’s post hoc analysis was utilized for analysis of echocardiographic and biochemical data. The statistical significance of oxylipin analyses was calculated by one-way ANOVA followed by a Tukey post hoc test. Results with *p* < 0.05 were considered significant. SPSS 15.0, SPSS version 24, and GraphPad Prism 5 were used to perform statistical computing.

## 3. Results

### 3.1. Cardiovascular Remodeling: Echocardiography

Echocardiographic parameters measured at baseline, including heart rate, interventricular septal wall thickness, posterior wall thickness, left ventricular end diastolic diameter (LVEDD), and LVEF, were comparable between all study groups (Table 1). Furthermore, interventricular septal wall thickness, posterior wall thickness, and heart rate remained within the normal physiologic range throughout the entire 6-week study.

**Figure 2:**
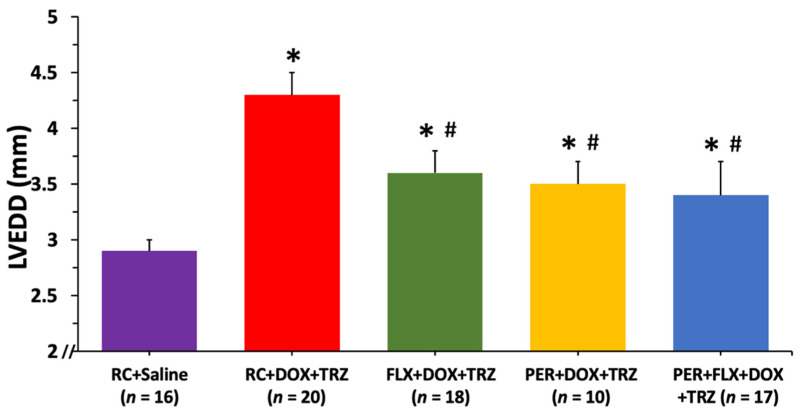
Changes in LVEDD values at week 6 compared to baseline in mice prophylactically administered FLX, PER, or FLX + PER treated with DOX + TRZ. Female C57Bl/6 mice treated with RC + DOX + TRZ experienced left ventricular cavity dilation as evident by an increase in LVEDD at week 6. Prophylactic treatment with FLX, PER, or FLX + PER significantly attenuated LV cavity dilation associated with DOX + TRZ administration. Data are expressed as mean ± SD. * *p* < 0.05 RC + DOX + TRZ vs. RC + saline. *^#^ *p* < 0.05 FLX + DOX + TRZ or PER + DOX + TRZ or PER + FLX + DOX + TRZ vs. RC + DOX + TRZ and RC + saline. DOX, doxorubicin; FLX, flaxseed; LV, left ventricle; LVEDD, left ventricular end diastolic diameter; PER, perindopril; RC, regular chow; SD, standard deviation; TRZ, trastuzumab.

There was a significant increase in LVEDD in mice treated with RC + DOX + TRZ, from 2.8 ± 0.2 mm at baseline to 4.5 ± 0.2 mm at study endpoint (*p* < 0.05). Prophylactically administering either FLX or PER alone diminished harmful left ventricular remodeling with LVEDD values of 3.6 ± 0.2 mm and 3.5 ± 0.2 mm at study endpoint, respectively (*p* < 0.05) (Figure 2). Administering FLX + PER concurrently did not provide synergistic cardioprotection at preventing left ventricular cavity dilation in mice treated with DOX + TRZ.

**Figure 3:**
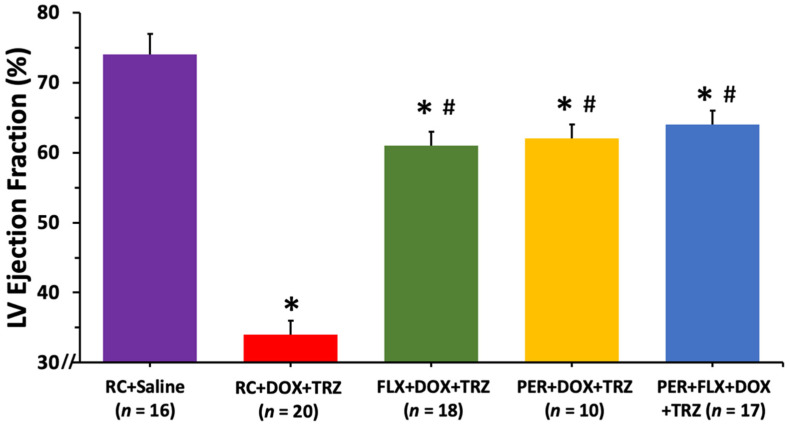
Changes in LVEF values at study endpoint in mice prophylactically administered FLX, PER, or FLX + PER treated with DOX + TRZ. Female C57Bl/6 mice treated with RC + DOX + TRZ experienced significantly impaired left ventricular systolic function as evident by a drop in LVEF at week 6. Prophylactic treatment with FLX, PER, or FLX + PER significantly improved the LVEF in animals administered DOX + TRZ. Data are expressed as mean ± SD. * *p* < 0.05 RC + DOX + TRZ vs. RC + saline. *^#^ *p* < 0.05 FLX + DOX + TRZ or PER + DOX + TRZ or PER + FLX + DOX + TRZ vs. RC + DOX + TRZ and RC + saline. DOX, doxorubicin; FLX, flaxseed; LV, left ventricle; PER, perindopril; RC, regular chow; SD, standard deviation; TRZ, trastuzumab.

Additionally, systolic function was impaired in mice treated with RC + DOX + TRZ as observed in an LVEF decline from 75 ± 2% at baseline to 34 ± 2% at week 6 (*p* < 0.05). The prophylactic use of either FLX or PER partially attenuated left ventricular systolic dysfunction with LVEF values of 61 ± 2% and 62 ± 2%, respectively (*p* < 0.05) (Figure 3). Prophylactically treating mice with the combination of PER + FLX, however, was not synergistic at protecting systolic function in mice treated with DOX + TRZ. As summarized in Table 2, there was little intra-observer and inter-observer variability for the LVEDD and LVEF measurements.

### 3.2. Biometrics and Histology: Electron Microscopy

**Figure 4:**
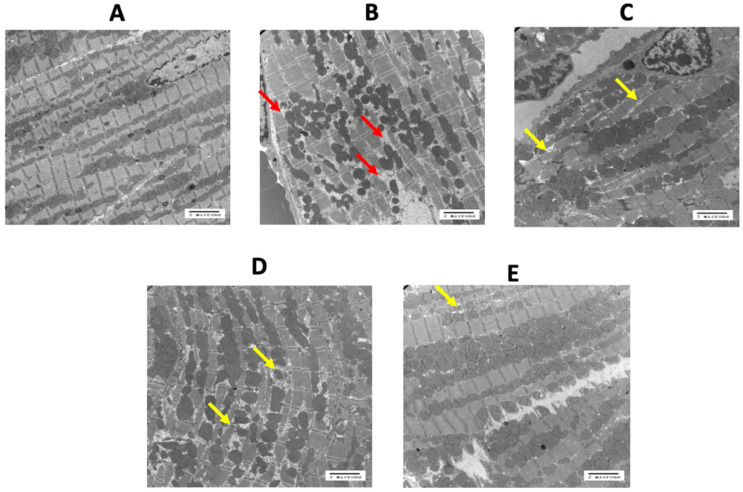
Ultrastructural changes in mice treated with DOX + TRZ prophylactically administered FLX, PER, or FLX + PER. Representative electron microscopy images of heart sample from C57Bl/6 female mice taken at 5800× magnification. (Panel **A**): RC + saline showcasing normal cellular integrity. (Panel **B**): RC + DOX + TRZ treatment led to severe damage and myofibril integrity (red arrows). Prophylactic treatment with FLX (Panel **C**), PER (Panel **D**), and FLX + PER (Panel **E**) partially prevented DOX + TRZ-mediated myocyte damage (yellow arrows). DOX, doxorubicin; FLX, flaxseed; PER, perindopril; RC, regular chow; TRZ, trastuzumab.

FLX and/or PER did not have an effect on body weight or heart weight at study endpoint. Electron microscopy, using a Philips CM 10 electron microscope, revealed normal cardiomyocyte structure in control mice (Figure 4A). In mice treated with RC + DOX + TRZ, there was a marked disruption of the myofibrils comprising the sarcomeres, as well as a loss of the sarcoplasmic reticulum (Figure 4B). Of note, mitochondria were not affected. Treatment with FLX, PER, or FLX + PER reduced these cellular changes (Figure 4C–E), as compared to treatment with DOX + TRZ (*p* < 0.05).

### 3.3. Plasma Oxylipin Concentrations

**Figure 5:**
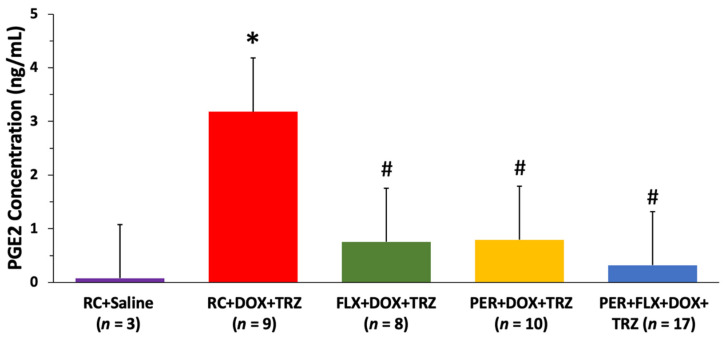
Changes in PGE2 concentrations in mice prophylactically administered FLX, PER, or FLX + PER treated with DOX + TRZ. C57Bl/6 female mice treated with RC + DOX + TRZ demonstrated a significant increase in the inflammatory oxylipin PGE2 at week 6. Prophylactic treatment with FLX, PER, or FLX + PER significantly attenuated elevations in PGE2 associated with DOX + TRZ administration. Data are expressed as mean ± SD. * *p* < 0.05 RC + DOX + TRZ vs. RC + saline. ^#^ *p* < 0.05 FLX + DOX + TRZ or PER + DOX + TRZ or PER + FLX + DOX + TRZ vs. RC + DOX + TRZ. DOX, doxorubicin; FLX, flaxseed; PER perindopril; PGE2, prostaglandin E2; RC, regular chow; SD, standard deviation; TRZ, trastuzumab.

As compared to the controls, mice treated with DOX + TRZ demonstrated a nearly 4-fold increase in the concentration of inflammatory oxylipins including prostaglandin E2 (*p* < 0.05). Prophylactic administration of FLX, PER, or FLX + PER, however, attenuated the elevations in these inflammatory oxylipins associated with DOX + TRZ (*p* < 0.05) (Figure 5).

### 3.4. Western Blotting: Inflammation

**Figure 6:**
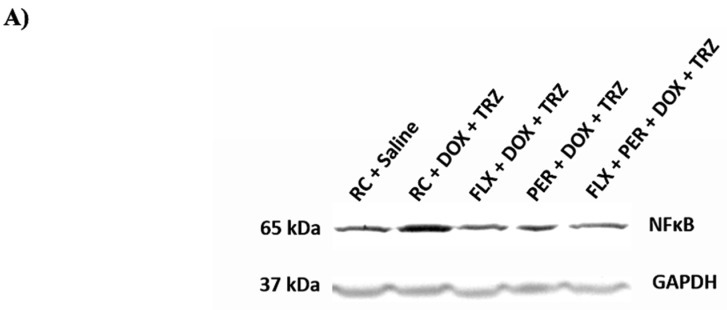

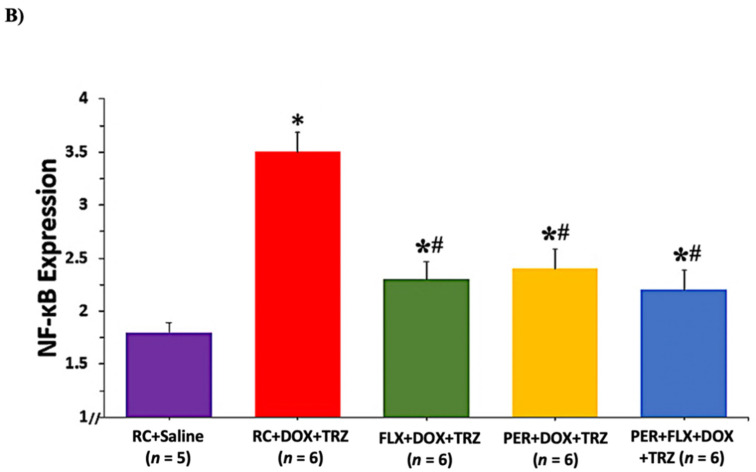
Changes in NF-kB expression in mice prophylactically treated with FLX, PER, or FLX + PER treated with DOX + TRZ. (**A**) Representative Western blot; (**B**) C57Bl/6 female mice treated with RC + DOX + TRZ demonstrated a significant increase in NF-κβ expression at week 6. Prophylactic treatment with FLX, PER, or FLX + PER significantly attenuated elevations in NF-κβ expression associated with DOX + TRZ administration. Data are expressed as mean ± SEM. * *p* < 0.05 RC + DOX + TRZ vs. RC + saline. *^#^ *p* < 0.05 FLX + DOX + TRZ or PER + DOX + TRZ, or FLX + PER + DOX + TRZ vs. RC + DOX + TRZ and RC + saline. DOX, doxorubicin; FLX, flaxseed; NF-κβ, nuclear factor kappa beta; PER, perindopril; RC, regular chow; SEM, standard error mean; TRZ, trastuzumab.

Through Western blotting techniques, excised cardiac tissues were assessed for NF-κB. At study endpoint, there was a 2-fold increase in NF-κB protein expression in DOX + TRZ-treated mice as compared to the RC + saline control (*p* < 0.05). Elevations in this inflammatory biomarker were significantly downregulated in mice prophylactically treated with FLX, PER, or FLX + PER (*p* < 0.05) (Figure 6 and Figure S1). 

## 4. Discussion

In 2022, cardiovascular disease and cancer remain the leading causes of death worldwide. Previous research has showcased the efficacy of nutraceutical agents, including FLX, and pharmaceutical agents, including PER, in reducing the risk of cardiac dysfunction resulting from anthracycline-based chemotherapy [8,9]. However, whether FLX is equivalent and/or synergistic to PER in the prevention of DOX + TRZ-mediated cardiotoxicity has yet to be explored.

Our novel findings illustrate that pre-treatment with dietary FLX is equivalent to PER at preventing cardiac dysfunction resulting from DOX + TRZ administration in a chronic in vivo female murine model of chemotherapy-induced cardiomyopathy. Specifically, the prophylactic use of either FLX or PER: (i) attenuated adverse left ventricular remodeling; (ii) ameliorated myofibrillar disarray; (iii) reduced inflammatory oxylipins; and (iv) decreased levels of the inflammatory biomarker NF-κB associated with DOX + TRZ treatment. However, the combined effects of FLX + PER did not exhibit synergistic cardioprotection.

### 4.1. Cardiovascular Remodeling

Several pre-clinical investigations have demonstrated adverse cardiovascular remodeling associated with DOX + TRZ administration. In an effort to accurately recapitulate current clinical practice, Milano et al. [24] developed a chronic in vivo murine model of DOX + TRZ-mediated cardiotoxicity. In the 6-week study, the administration of a cumulative dose of 24 mg/kg DOX followed by 10 mg/kg TRZ resulted in severe cardiotoxicity in mice while maintaining high survival rates. In comparison to the control, DOX + TRZ treatment resulted in significant LV cavity dilation and LV systolic dysfunction, leading to a dilated cardiomyopathy (*p* < 0.05) [24]. Corroborating these findings, pre-clinical studies performed by our research group have demonstrated that prophylactic treatment with RAS antagonists, antioxidants, or nutraceuticals were all partially cardioprotective at preventing adverse cardiovascular remodeling [5,8,9]. In the present study, treatment with DOX + TRZ resulted in a 1.5-fold increase in LV cavity dilatation and a 2-fold decrease in LV systolic function at study endpoint. Prophylactic administration with either FLX or PER significantly attenuated adverse cardiovascular remodeling in our study. The cardioprotective benefits of FLX + PER, however, did not appear to have additive effects at preventing adverse LV remodeling due to DOX + TRZ.

### 4.2. Histologic Analysis

In addition to the phenotypic development of a dilated cardiomyopathy, microscopic loss of cardiomyocyte integrity is a well-documented consequence of DOX + TRZ treatment [8,9,28,29,30]. Mitochondrial swelling, vacuolization of the cytoplasm, dilation of the sarcotubular system, and formation of lysosomal bodies are well documented ultrastructural manifestations in rats treated with DOX over a 2-week period [8,9,31,32]. Laird-Fick et al. [33] demonstrated that myofiber necrosis, rare apoptosis, and perivascular infiltration of macrophages is associated with TRZ administration in rabbits. In addition, treatment with both DOX + TRZ has induced more severe ultrastructural changes as compared to either therapy alone [4,8,10,12,19]. Our current findings are consistent with the aforementioned studies, whereby treatment with DOX + TRZ resulted in significant ultrastructural changes, including a loss of myofibril assembly and increased cytoplasmic vacuolization. Our study was notable for the combined effects of FLX + PER in preserving cardiomyocyte ultrastructure as compared to the control.

### 4.3. Inflammation

The role of inflammation, leading to cardiac fibrosis, apoptosis, and ultimately heart failure, has been proposed as a central mechanism of DOX + TRZ-mediated cardiotoxicity [5,6,9]. Through the extensive and variable oxidation of polyunsaturated fatty acids, oxylipins have emerged as a key bioactive mediator of human physiology and inflammation [34]. Similarly, determining tissue concentrations of molecular biomarkers including NF-κB remains central to increasing awareness of the effects of inflammation on cardiac homeostasis in the setting of DOX + TRZ-mediated cardiotoxicity.

Cyclooxygenase-derived oxylipins, including prostaglandin E2, are key regulators in physiologic and pathologic inflammatory responses [35,36,37,38,39]. Previous studies performed by our research group have shown a significant rise in the concentration of this cyclooxygenase-derived oxylipin in a chronic in vivo model of DOX + TRZ-mediated cardiotoxicity, with FLX and PER displaying cardioprotection [6,8,9]. The results from the current study are consistent with prior studies, whereby prophylactic administration of FLX or PER was able to significantly attenuate elevations in the inflammatory oxylipin prostaglandin E2 (*p* < 0.05). While the prophylactic use of either FLX or PER reduced the upregulation of these inflammatory oxylipins, the combined effects of FLX + PER were not synergistic. With the diverse and interconnected functions of oxylipins still being explored, additional studies providing further insights into their specific role in the setting of DOX + TRZ-mediated cardiotoxicity are warranted.

Finally, a central component of DOX + TRZ-mediated cardiotoxicity involves the up-regulation of pro-inflammatory mediators, including NF-κB [4,5,7,8,9]. Once activated, NF-κB serves to modulate cytokine production and cell survival [4,8]. A pre-clinical investigation conducted by Akolkar et al. [7] demonstrated a 1.5-fold increase in NF-κB expression associated with chronic DOX administration in an in vivo animal model. A more recent study performed by our lab documented a 2.0-fold increase in myocardial NF-κB in a chronic in vivo female murine model of DOX + TRZ-mediated cardiotoxicity [9]. Interestingly, in this 6-week study, inflammatory biomarkers were significantly attenuated in animals pre-treated with the nutraceutical agent FLX [8]. The results from our current study corroborate the aforementioned investigations, whereby prophylactic administration with either FLX or PER alone was able to reduce elevations in NF-κB concentrations observed in mice treated with DOX + TRZ. Importantly, modulation of the NF-κB signalling pathway has also been implicated in the prevention and treatment of breast cancer [40]. The inhibition of the NF-κB signalling pathway has been shown to increase cancer cell responsiveness to anti-cancer therapies, further highlighting the potential benefit of FLX and PER among individuals with breast cancer [40].

## 5. Conclusions

In a chronic in vivo female murine model, we demonstrated that the prophylactic use of either FLX or PER was equivalent at partially attenuating the cardiotoxic side effects resulting from DOX + TRZ administration. 

## Figures and Tables

**Table 1:** Echocardiographic parameters at baseline and 6 weeks.

Variable	Group	Baseline	Week 6	*p* Value
HR (beats per minute)	RC + saline (*n* = 16)	694 ± 6	690 ± 7	0.84
	RC + DOX + TRZ (*n* = 20)	687 ± 9	693 ± 6	0.81
	FLX + DOX + TRZ (*n* = 18)	693 ± 5	690 ± 4	0.82
	PER + DOX + TRZ (*n* = 10)	688 ± 7	692 ± 5	0.71
	FLX + PER + DOX + TRZ (*n* = 17)	691 ± 4	689 ± 3	0.82
PWT (mm)	RC + saline (*n* = 16)	0.81 ± 0.01	0.81 ± 0.02	0.99
	RC + DOX + TRZ (*n* = 20)	0.82 ± 0.02	0.82 ± 0.01	0.98
	FLX + DOX + TRZ (*n* = 18)	0.82 ± 0.01	0.81 ± 0.02	0.92
	PER + DOX + TRZ (*n* = 10)	0.82 ± 0.02	0.82 ± 0.01	0.98
	FLX + PER + DOX + TRZ (*n* = 17)	0.82 ± 0.01	0.82 ± 0.02	0.97
LVEDD (mm)	RC + saline (*n* = 16)	2.8 ± 0.1	2.9 ± 0.1	0.78
	RC + DOX + TRZ (*n* = 20)	2.8 ± 0.1	4.5 ± 0.2 *	<0.05
	FLX + DOX + TRZ (*n* = 18)	2.8 ± 0.2	3.6 ± 0.2 *^#^	<0.05
	PER + DOX + TRZ (*n* = 10)	2.8 ± 0.1	3.5 ± 0.2 *^#^	<0.05
	FLX + PER + DOX + TRZ (*n* = 17)	2.8 ± 0.2	3.4 ± 0.3 *^#^	<0.05
LVEF (%)	RC + saline (*n* = 16)	74 ± 2	74 ± 3	0.92
	RC + DOX + TRZ (*n* = 20)	75 ± 2	34 ± 2 *	<0.05
	FLX + DOX + TRZ (*n* = 18)	73 ± 4	61 ± 2 *^#^	<0.05
	PER + DOX + TRZ (*n* = 10)	74 ± 3	62 ± 2 *^#^	<0.05
	FLX + PER + DOX + TRZ (*n* = 17)	74 ± 3	64 ± 2 *^#^	<0.05

All echocardiographic parameters were assessed weekly throughout the study period. The values are presented as mean ± SD. * *p* < 0.05 RC + DOX + TRZ vs. RC + saline. *^#^ *p* < 0.05 FLX + DOX + TRZ or PER + DOX + TRZ or PER + FLX + DOX + TRZ vs. RC + DOX + TRZ and RC + saline. DOX, doxorubicin; FLX, flaxseed; HR, heart rate; LVEDD, left ventricular end-diastolic diameter; LVEF, left ventricular ejection fraction; PER, perindopril; PWT, Posterior wall thickness; RC, regular chow; SD, standard deviation; TRZ, trastuzumab.

**Table 2:** Intra- and inter-observer variabilities of the echocardiographic parameters.

Echocardiographic Parameters	Mean Difference ± Standard Deviation
Intra-observer variability	
LVEDD, mm	0.03 ± 0.01
LVEF, %	0.5 ± 0.2
Inter-observer variability	
LVEDD, mm	0.04 ± 0.01
LVEF, %	1.1 ± 0.2

Values are mean ± SD (*n* = 25 mice).

## Data Availability

The datasets used and/or analyzed during the current study are available from the corresponding author upon reasonable request.

## Supplementary Materials

The following supporting information can be downloaded at: https://www.mdpi.com/article/10.3390/curroncol29050241/s1. Figure S1: The raw western blot film used in the assessment of NF-kB (65 kDa) normalized to GAPDH (35 kDa) loading control.

## Abbreviations

ACEi	Angiotensin-converting enzyme inhibitor
DOX	Doxorubicin
FLX	Flaxseed
HER2	Human epidermal growth factor 2
LVEDD	Left ventricular end diastolic diameter
LVEF	Left ventricular ejection fraction
NF-κβ	Nuclear factor kappa beta
PER	Perindopril
RAS	Renin angiotensin system
RC	Regular chow
TRZ	Trastuzumab
