# Supplementary material for: Comparing Flaxseed and Perindopril in the Prevention of Doxorubicin and Trastuzumab-Induced Cardiotoxicity in C57Bl/6 Mice
Source: Curr Oncol. 2022 Apr 21;29(5):2941–53. doi: 10.3390/curroncol29050241 (PMC9139942; doi:10.3390/curroncol29050241)
Supplement: Supplementary file 1 [file curroncol-29-00241-s001.zip › curroncol-1662320-supplementary.pdf]

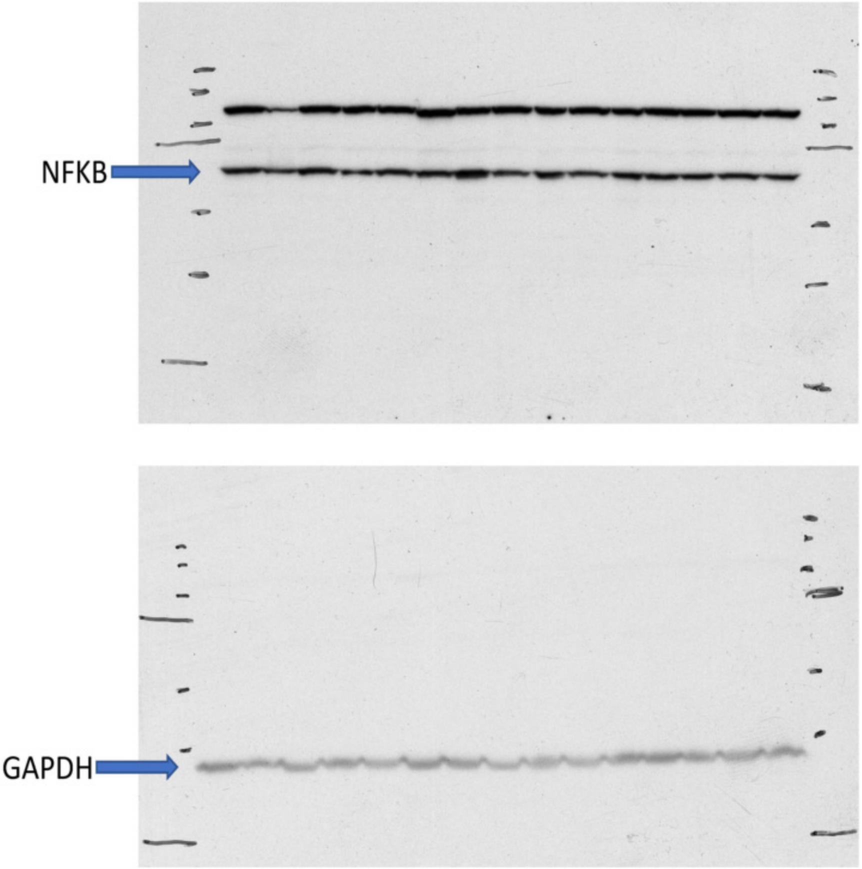

Figure S1. The raw Western blot film used in the assessment of NF-kB (65kDa), normalized to GAPDH (35kDa) loading control.
